# 

**Published:** 1915-04-17

**Authors:** 


### CONTENTS.

PAGE

**The National Care of the Sick and Wounded** 45

**The War Office and its Temporary Hospitals...** 46

**Hospital and Institutional News ... ... ...** 47

Sir Alfred Keogh and the Need for Doctors—THE HOSPITAL'S Plea for Part-Time Service—Panel Chemists and their Accounts—Vacant Medical Posts not Filled—Hospital Authorities and Inappropriate Advertisements—The Difficulties Due to Small Poor-Law Areas—The Example of Birmingham—Tribute to the Ex-Chairman of the Saturday Fund—The "Tigers'" Bed at Leicester—Wounded Soldiers in Poor-Law Institutions—Summer Frost-Bite: A French View—The Salaries of Tuberculosis Officers—An Overworked Doctor—Wounded from the Dardanelles—An Architect as Hospital Secretary—Insured Persons in Poor-Law Infirmaries—A Remarkable Extension at Nottingham—Relief Hospitals and Convalescent Homes—An Octogenarian's Gift—Paying Patients in Cottage Hospitals—Winsley Sanatorium and the New Scheme—The Leeds Extension—Alcohol in Patent Medicines—The New Commissioner for Red Cross Relief in Serbia—This Week's Drug Market—To Our Readers.

PAGE

**The Comforts and Feeding of the Wounded During Transit ... ... ... ... ...** 53

A Review of the Supply Organisation.

By Sir George Beatson, K.C.B.

**A Red Cross Field Soup Kitchen ... ... ...** 56

The Design and Equipment in Detail.

**Buildings Contemplated ... ... ... ...** 56

**The Overseas Transport of Wounded ... ...** 57

Life and Procedure on a Hospital Ship.

By a Medical Officer on the Ambulance Ship *St. David*.

**Organisation of a Red Cross Train ... ...** 59

The Transit by Rail.

By an R.A.M.C. Officer in Charge.

[*See over.*]

### CONTENTS—(*continued*).

PAGE

**Organising a Voluntary Aid Detachment** ... 61
The Last Link in the Transport Service.
By A. W. Faire, County Director of Leicester Territorial Association.

**The Legal Status of the Hospital Ship in War-Time** ... ... ... ... ... ... 63

**Hospitals' Liability and Cases of Negligence** ... 63

**Round the World** ... ... ... ... ... 64

Fellow-Workers and the Roll of Honour.

**A Medical Causerie** ... ... ... ... ... 65

**The Institutional Library** ... ... ... ... 66
Practical Points on Open-Air Schools.
The Theory and Practice of After-Care.
The Medical Woman's Opportunity.

**Building a Hospital in India** ... ... ... 67
I.—The Story of the Karnal District Hospital.
By Major C. H. Buck, I.A., Deputy Commissioner.

PAGE

**The Evolution of the Modern Hospital** ... ... 69
VII.—The Problem of the Unpaid Staff.
By B. Burford Rawlings.

**Food in War-Time** ... ... ... ... ... 71

**The Editor's Letter-Box** ... ... ... ... 72

How to Utilise Fat and Fish-Bones?

The After-Care of the Wounded.

**Institutional Needs** ... ... ... ... ... 72

**Gifts and Bequests** ... ... ... ... ... 76

**The Institutional Worker** ... ... (Suppl.) 1
IV.—The Almoner.
Questions for April.
Questions Invited.
Noise in Institutions.
Enquiries and Answers.
Employment and Training.
The Sick and in Need.

